# Fabrication of Definitive Cast for Complete Mouth Rehabilitation by Using Resin-Transfer Coping

**DOI:** 10.7759/cureus.27831

**Published:** 2022-08-09

**Authors:** Saeed J Alzahrani

**Affiliations:** 1 Restorative Department, King Abdulaziz University, Jeddah, SAU

**Keywords:** full-mouth rehabilitation, dentate rehabilitation, fixed prosthodontics, final impression, master cast

## Abstract

One of the most challenging steps in dentistry is taking final impressions for full mouth rehabilitation of a dentate patient. Final impressions should include the necessary details needed to fabricate a definitive cast. This cast will be used to construct indirect restorations in the laboratory. Besides all the improvement of impression materials and systems used for taking final impressions, the intraoral cavity is a challenging environment and there are some cases that prevent clinicians from recording the needed details in a single final impression. The objective of this case report is to describe a technique for fabrication of a definitive cast for a full mouth rehabilitation case by using resin-transfer coping impression technique. This technique is used as an alternative option for cases that prevent the clinician from capturing all the needed details in a single full-arch final impression.

## Introduction

Full mouth rehabilitation is indicated for many reasons, one of the most common reasons is worn dentition [1]. There are several etiologies that can lead to excessive occlusal wear, such as congenital anomalies like amelogenesis and dentinogenesis imperfecta, parafunctional habits such as bruxism, abrasion such as chewing tobacco, or combination of multiple etiological factors [2]. Turner et al. classified occlusal wear into three categories [3]. These categories are: excessive wear with loss of occlusal vertical dimension, excessive wear without loss of occlusal vertical dimension but with space available, and excessive wear without loss of occlusal vertical dimension but with limited space.

Fabrication of a definitive cast is one of the most important steps in full mouth rehabilitation. This step prerequisites an accurate final impression [4]. Intraoral cavity is a challenging environment and requires meticulous handling from the clinician to obtain an accurate final impression [5]. Accurate definitive cast is completeness of the accurate impression and, thus, definitive cast must meet certain requirements like recording of the prepared tooth and unprepared structure apical to the finish line and involvement of adjacent teeth and surrounding soft tissue in the design of the prosthesis [6]. Final impression for full mouth rehabilitation on natural teeth is a difficult procedure and becomes complex when the patient is suffering from obesity (thick buccal checks), tongue-tie and limited mouth opening. This dilemma faced multiple clinicians, and there are techniques suggested to overcome these situations [7,8]. Gardener and Loft presented an intraoral coping technique [7]. First, resin shell is made on duplicate of the wax-up cast. After teeth preparations, the resin shell is relined with resin, then relieved from inside to be filled with impression for each abutment. After relining every single coping with impression, all the relined copings seated back intraorally to each belonging abutment and all over impression were taken. Donavan and Chee suggested the segmental impression technique [8]. This technique divided the arch into multiple segments and segmental custom trays are made for each area of the arch. Then, the impression of every segment is taken separately. After taking an impression for each segment, all the segmental impressions are placed back intraorally, and a pick-up impression is taken.

Resin-transfer coping has been suggested to be used for many purposes in fixed prosthodontics, like when single or multiple teeth are not captured accurately for full-arch impression [9]. In this technique, the clinician evaluates the master cast and retakes the final impression for only the teeth missing the needed details (finish line, unprepared tooth structure below finish line, soft tissue surrounding abutment and neighboring teeth) to fabricate the final prosthesis. After that, the multiple master casts are joined into one cast by using resin-transfer coping. It is also used to insert the implant’s abutment in the patient's mouth [10]. In this, transfer coping on top of the implant’s parts at the definitive cast is fabricated to precisely transfer implant’s abutment from the definitive cast to the patient's mouth and save the clinician's time. Additionally, it makes the teeth-reduction guide on definitive cast before fabrication of the final prosthesis [11]. After pouring the impression and mounting the definitive cast, lab technicians might find that the abutment preparation is not enough to fabricate the prosthesis for esthetic or functional reasons. For this purpose, resin coping was fabricated on the definitive cast’s abutment. Then, the lab technician prepared through the resin coping and abutment to obtain required esthetic or functional space to fabricate the prosthesis. After that, this prepared coping over the abutment will be sent to the clinician as guide to prepare the teeth as required. Moreover, it is also used to make interocclusal records for prepared teeth [12]. Many clinicians prefer to register interocclusal records with rigid and stable material as the resin because other interocclusal materials might cause error in the mounting (polyvinyl silicon bite-registration materials have elastic behavior and wax bite-registration materials have thermoplastic changes). The objective of this article is to describe a technique for fabrication of a definitive cast by using resin-transfer coping for a patient complaining of worn dentition, treated with full mouth rehabilitation.

## Case presentation

**Figure 1:**
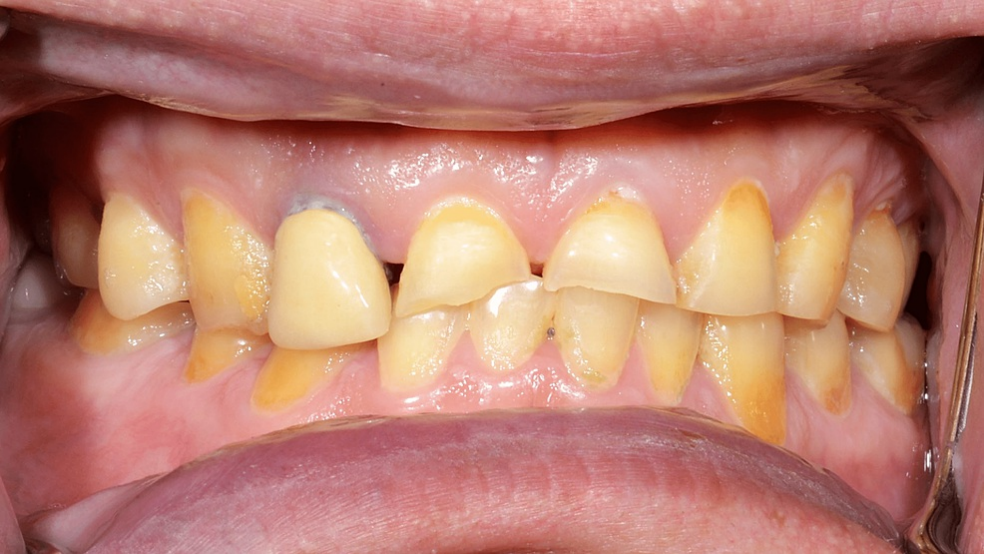
Initial presentation

A 49-year-old male patient presented to the clinic complained of worn dentition (Figure 1). The etiology of this occlusal wear is parafunctional habits. Patient declared that due to occupational and emotional stresses, he used to grind his teeth. He reported no systemic disease except obesity and did not report any medications taken. Extraoral examination showed no muscle tenderness, no palpable lymph nodes. Mandibular range of motion was smooth and free of clicking, and there was no deviation nor deflection of the mandible upon opening and closing.

**Figure 2:**
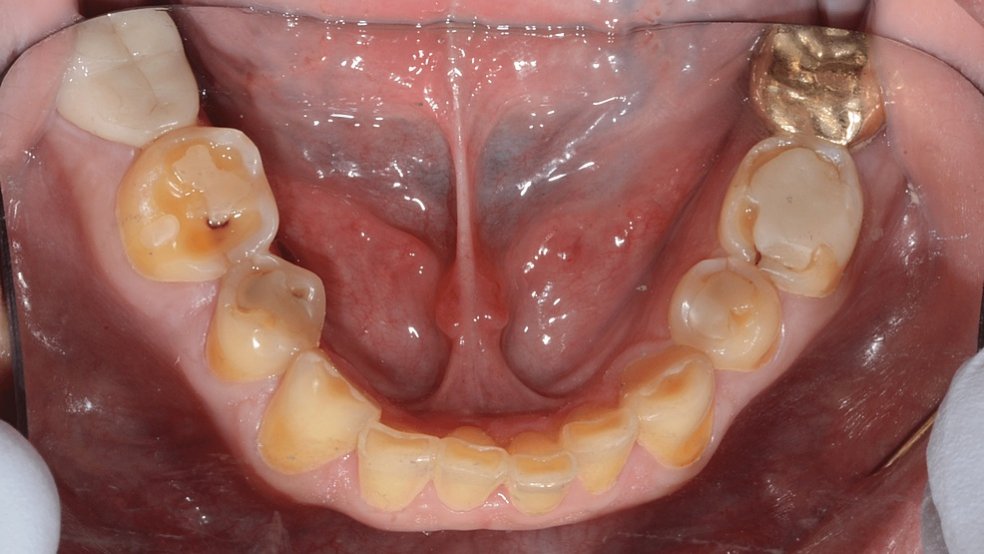
High lingual frenum attachment

Intraoral examination revealed that the hard and soft palate, buccal mucosa, and oropharynx are within normal limits. Patient has higher lingual frenum of the tongue which prevented full motion of the tongue (Figure 2).

**Figure 3:**
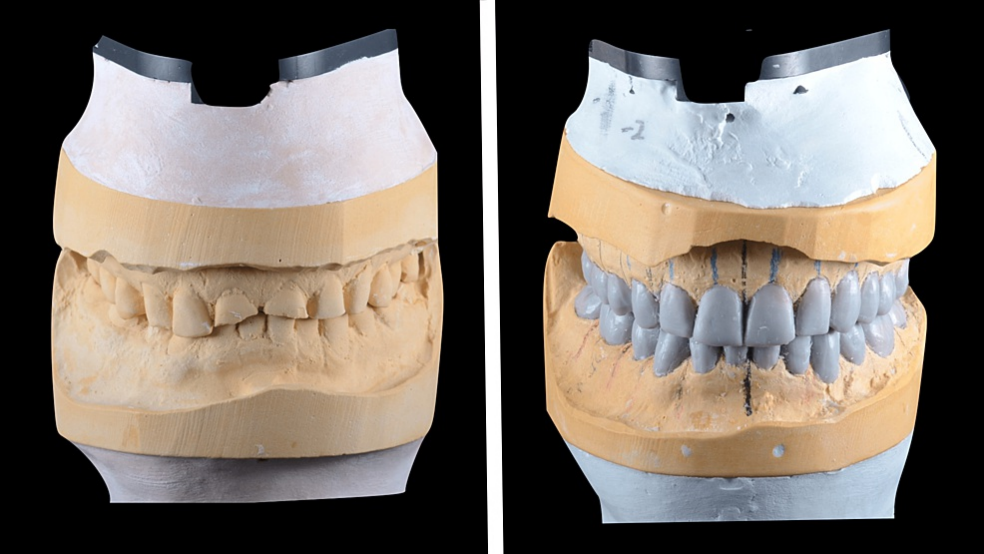
Study and wax-up casts

Saliva was normal in flow and consistency. The patient stated that due to crowding when he was young, his orthodontist did a comprehensive orthodontic treatment and extracted maxillary and mandibular first premolars to resolve this issue. Also, the oral surgeon had extracted all third molars. The patient's existing vertical dimension was assessed and there was 5 mm of interocclusal rest space. A Facebow record was taken with Denar® Slidematic Facebow to mount the maxillary cast on the Denar Mark II articulator. For esthetic and restorative material, centric relation was recorded with 2 mm increased in vertical dimension at occlusion. The interocclusal records were used to articulate the mandibular cast to maxillary cast on the proposed vertical dimension. The right and left horizontal condylar inclinations were set to 20 degrees. The lateral condylar inclinations were set to 15 degrees. Diagnostic wax-up was completed to plan the anticipated occlusion and to aid in the evaluation of the potential esthetic outcome (Figure 3).

**Figure 4:**
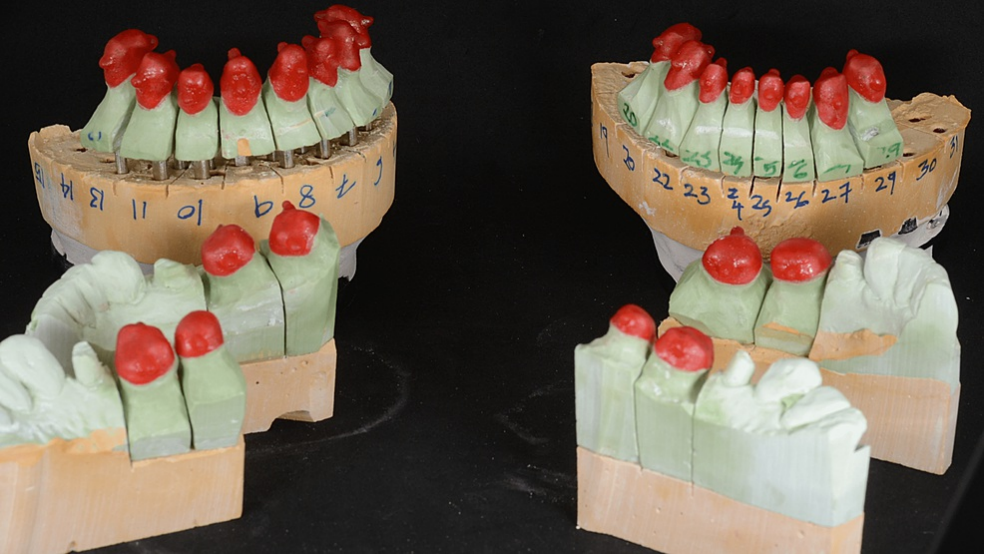
Resin-transfer coping on dies

Treatment plan discussed with the patient in detail included expectations, limitations, costs, risks and benefits, number of visits, length of treatment, and procedures. Different treatment options also were discussed in detail including the type of the materials (full ceramic, zirconia, layered zirconia, and metal ceramic) and design of the prosthesis (partial coverage or full coverage). Following a review of all treatment options, objectives, and limitations, the definitive treatment plan formulated was a full mouth rehabilitation with 2 mm increase in vertical dimension to restore the esthetic and function. Due to the size of the lower anteriors, a conservative preparation for full coverage heat-pressed lithium disilicate restorations on teeth #33, 32, 31, 41, 42, and 43 were done. Due to patient’s heavy occlusal forces and masking metal core on teeth #11, 21, and 22, full coverage metal-ceramic restorations were chosen to restore the remaining teeth. After four weeks of wearing the occlusal orthotics to evaluate and test the acceptance of the patient to a new vertical dimension. Patient accepted the new vertical dimension of occlusion with no complaint. Full mouth teeth preparations were performed in a single visit. Then, lab-processed polymethyl-methacrylate provisional shells were relined, finished, polished and cemented with zinc oxide non-eugenol cement. The patient wore interim restorations for a total of two months and was happy with the esthetics, phonetics, occlusion, and comfort. Due to thick buccal checks, high frenum attachment of the tongue, and limited mouth opening, it was difficult to make a full-arch final impression. The plan was to divide the arch into three segments: anterior and premolars teeth segment, left molars teeth segment, and right molars teeth segment. Each segment's final impression was taken separately by using double-cord technique [13] and prepared using light- and heavy-body polyvinyl siloxane impression material (Aquasil; Dentsply Intl, York, USA). Type V high-strength high-expansion die stone (Hard Rock; Whip Mix Corp, Louisville, USA) was poured into the impressions. Removable dies were made with the dowel pins. Then, a layer of separating medium was applied on all dies to fabricate resin-transfer coping on each die. The resin material was pattern resin (Pattern Resin; GC, Alsip, USA) and the manual technique of layering powder and liquid with brush was used (Figure 4).

**Figure 5:**
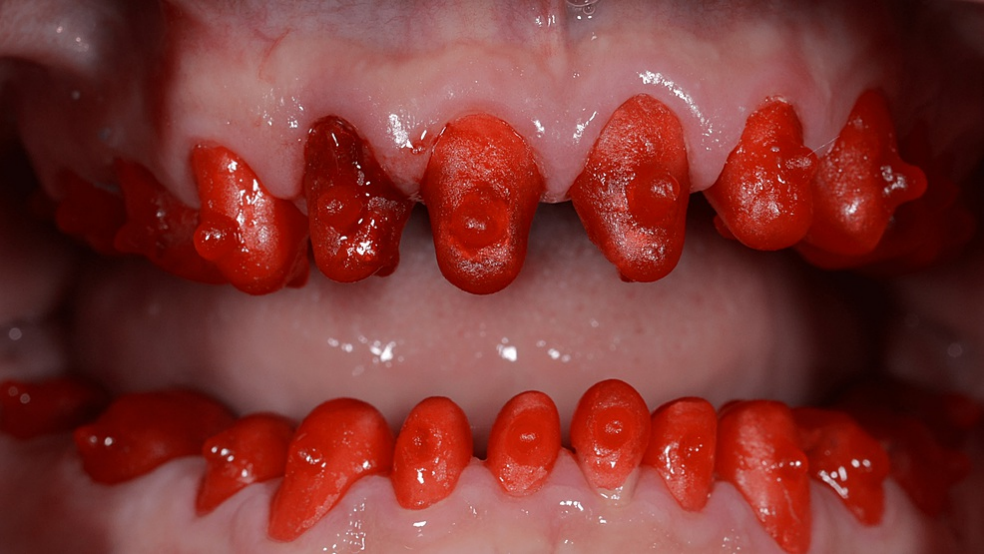
Transfer resin copings in situ

Resin coping was transferred to the patient's mouth and seated on each belonging abutment. To enhance bonding to the impression material during coping pick-up, an adhesive layer was applied on the seated coping (Caulk tray adhesive, Dentsply Intl, York, USA) (Figure 5).

**Figure 6:**
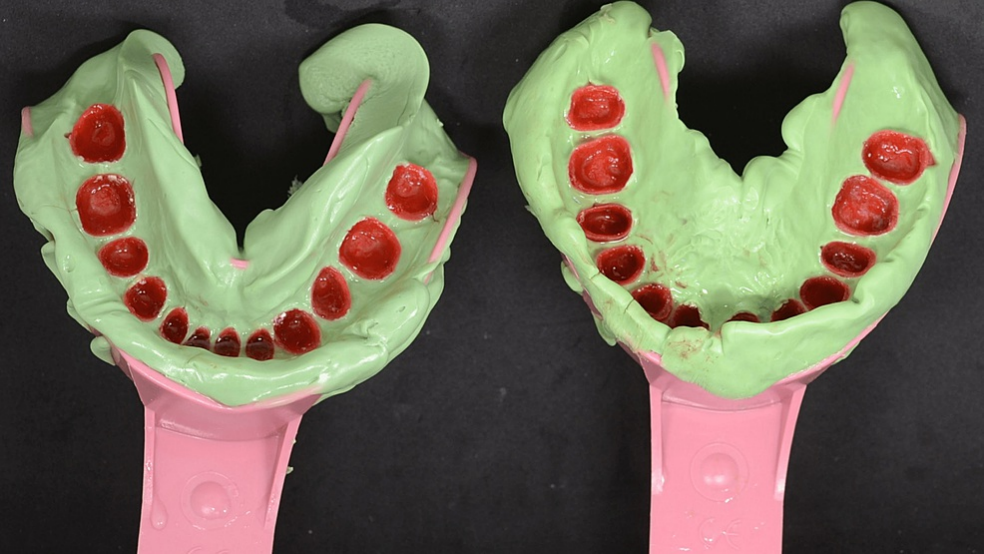
Maxillary and mandibular pick-up impressions of resin-transfer coping

**Figure 7:**
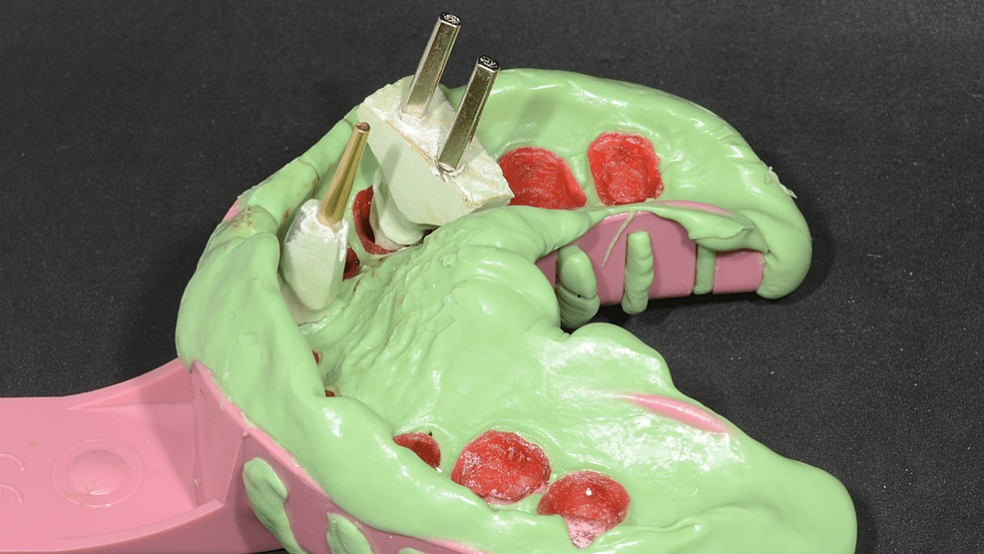
Die seated in the resin-transfer coping

A pick-up impression was taken using heavy-body impression material (Aquasil) (Figure 6). After all the resin-transfer coping were picked up with the impression, dies were modified to be seated into the belonging resin-transfer coping (Figure 7).

**Figure 8:**
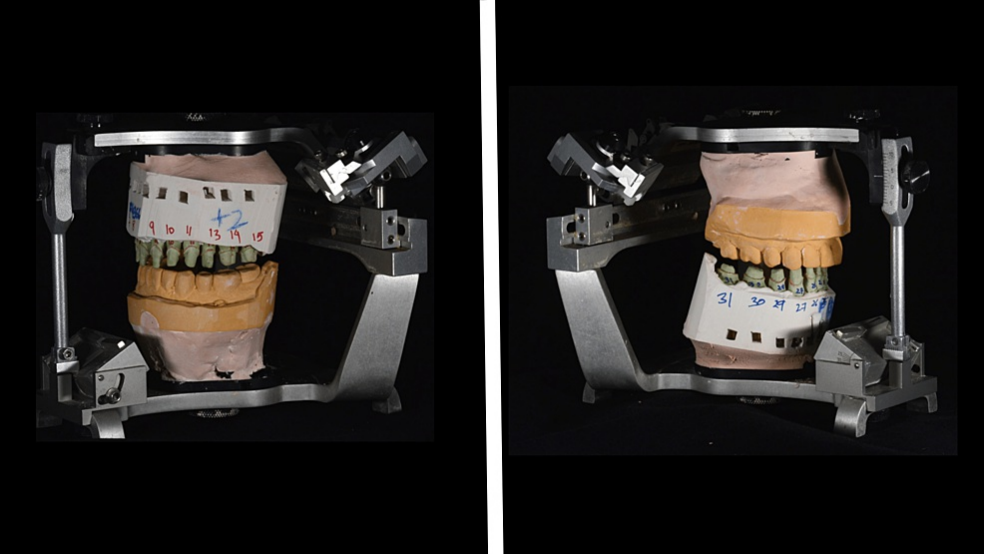
Cross-mounted definitive casts

A layer of separating medium on dies was applied before before Type IV low expansion die stone (Silky Rock, Whip Mix Corp, Louisville, USA) was poured. After pouring, the master cast was ready to be mounted on the articulator and cross-mounted to start fabrication crowns as planned (Figure 8).

**Figure 9:**
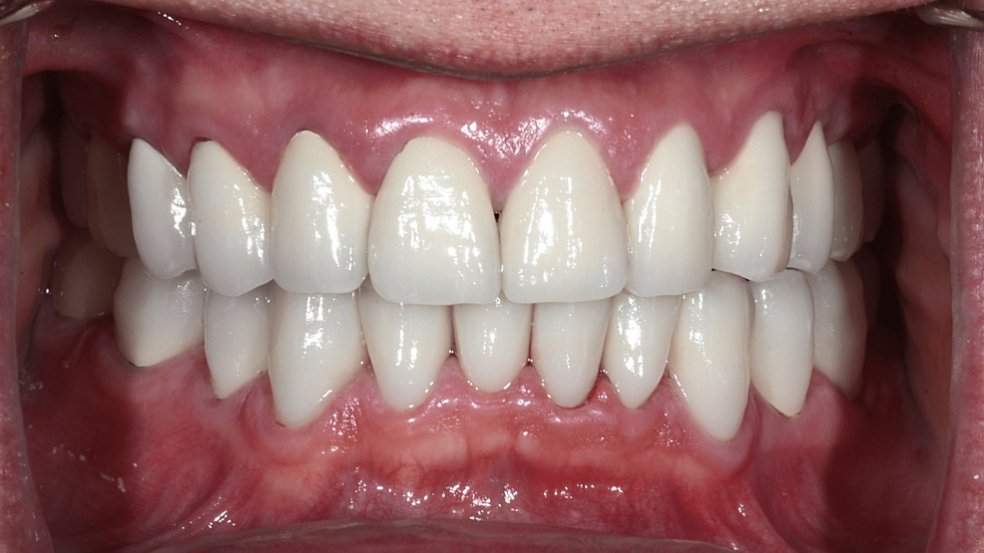
Prosthesis in situ

**Figure 10:**
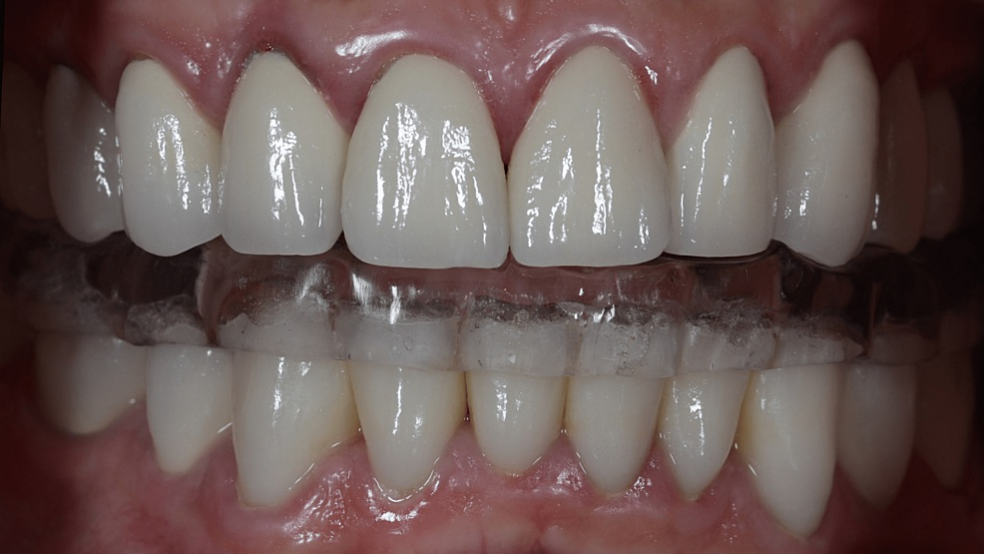
Night guard in situ

A metal framework try-in was done to check the seating and fit of the castings (Argelite 55; Dentalloy, Iserlohn, Germany). Also, articulator-jaw relation was verified during the try-in. Then, porcelain bisque crowns try-in was accomplished (Noritake Super porcelain EX-3, Kuraray America Inc., New York, USA). Interproximal contacts were checked and adjusted as needed using shim stock and confirmed with floss. The occlusion was checked and modified as needed. After glazing, abutments were cleaned of any debris of remaining temporary cement. Resin cement was used for cementation of all-ceramic restorations, and resin-modified glass ionomer cement for metal-ceramic restorations. Excess cement removed with floss, scalers, and an explorer. Mutually protective occlusal scheme was achieved (Figure 9). Post-operative instruction and maintenance protocols were given. A night guard was inserted after 24 hours (Figure 10). The patient was followed up at one-week, one-month, and then at six-month intervals for three years. He was very comfortable and satisfied with the esthetic and functional outcomes of the treatment.

## Discussion

Single full-arch impression is the aim for any clinician to fabricate definitive casts in fixed prosthodontics. A dry field should be achieved and maintained before tissue displacement (chemical or mechanical) and taking final impression. A number of techniques were suggested to avoid moisture contamination during final impression registration such as placing cotton rolls, saliva ejector, saliva evacuator, rubber dam for supragingival finish line, and absorbent card. However, there are many cases where moisture contamination is unavoidable. In this presented case, two attempts were made to capture all the needed details in a single final impression but, unfortunately, thick buccal mucosa, tongue-tie, and limited mouth opening (> 25 mm) prevented us from achieving appropriate final impressions. All the finish lines on the abutments were also located subgingivally for esthetic and function reasons, which added to the difficulty. For the maxilla, maxillary rolls do not stay in position in both vestibules and the only way to retain them was with a finger or the mouth mirror. In addition, Buccal mucosa retraction can only be done at one side at a time due to the thick buccal mucosa. For the mandible, the cotton roll on the buccal side slips during retraction, and due to tongue-tie, limited tongue movement does not allow placement of cotton rolls underneath the tongue to block off the sublingual and submandibular salivary ducts. Attempt was made to use the intraoral scanner, but the patient's mouth opening could not allow the scanner to scan posterior teeth and rotate scanner buccally and lingually easily. Gardner and Loft's technique [7] needs more clinical time to be able to reline the shell and relieve space for the impression material. Donovan and Chee's technique [8] requires a good mouth opening to be able to pick up all the segmented impressions in one impression. Another way to solve this issue is by mounting every segmented master cast and fabricating the frameworks (metal or ceramic). Then after try-in, all the framework must be picked up in impression to have one master cast. But this technique requires multiple mountings, and the accuracy of fabricating the frameworks will be questionable due to discrepancy between the mounting of segmented impressions. The disadvantage of the presented technique in this case report is that it requires meticulous handling by the clinicians and dental lab technician in each step to avoid repetition. The limitation was unavailability of intraoral scanner with a smaller size tip to scan the teeth. 

## Conclusions

Definitive cast is an important step for fabrication dental prosthesis. The aim of this case report was to describe a technique for fabrication of a definitive cast for a full mouth rehabilitation case by using resin-transfer coping impression technique. The presented technique is used as an alternative option for cases that prevent the clinician from capturing all the needed details in a single full-arch final impression.
